# Genome rearrangements with indels in intergenes restrict the scenario space

**DOI:** 10.1186/s12859-016-1264-6

**Published:** 2016-11-11

**Authors:** Laurent Bulteau, Guillaume Fertin, Eric Tannier

**Affiliations:** 1 Laboratoire d’Informatique Gaspard Monge, CNRS UMR 8049, Université Paris-Est, 5 Bd Descartes, Marne-la-Vallée, 77454 France; 2 LINA UMR CNRS 6241, Université de Nantes, 2 rue de la Houssinière, Nantes, 44322 France; 3 Laboratoire de Biométrie et Biologie Évolutive (LBBE), 43 boulevard du 11 novembre 1918, Villeurbanne, 69622 France; 4 Institut National de Recherche en Informatique et en Automatique (INRIA) Rhône-Alpes, 655 avenue de l’Europe, Montbonnot-Saint-Martin, 38330 France

**Keywords:** DCJ, Intergenic regions, Indels, Genome rearrangements

## Abstract

**Background:**

Given two genomes that have diverged by a series of rearrangements, we infer minimum Double Cut-and-Join (DCJ) scenarios to explain their organization differences, coupled with indel scenarios to explain their intergene size distribution, where DCJs themselves also alter the sizes of broken intergenes.

**Results:**

We give a polynomial-time algorithm that, given two genomes with arbitrary intergene size distributions, outputs a DCJ scenario which optimizes on the number of DCJs, and given this optimal number of DCJs, optimizes on the total sum of the sizes of the indels.

**Conclusions:**

We show that there is a valuable information in the intergene sizes concerning the rearrangement scenario itself. On simulated data we show that statistical properties of the inferred scenarios are closer to the true ones than DCJ only scenarios, i.e. scenarios which do not handle intergene sizes.

## Background

In a previous publication [1], we have argued that intergenic sizes were a crucial parameter to infer genome rearrangement distances. Indeed, ignoring this information, as all published distance estimations were doing so far [2], leads to strong biases in all estimations and validation procedures. Here we explore the information contained in intergene size distributions, not for rearrangement distances but for rearrangement *scenarios*. We use a weighted DCJ operation that acts both on gene order and intergene sizes [3]. In addition, in order to account for all the size variations in intergenic regions, we introduce the possibility of performing indels in intergenes.

We present a polynomial-time algorithm that reconstructs a DCJ scenario which optimizes on the number of DCJs, and given this optimal number of DCJs, optimizes on the total size of the indels. We use it to restrict the solution space of rearrangement scenarios. Indeed it is known that such a space is huge [4, 5], which makes it hard to analyze; several methods have thus been devised to add genomic or epigenomic constraints to restrict the search space [6–8]. So far, the potential of intergenic sizes has only been explored for distance computations [1, 3]. We show that it can also contain information on the scenarios, by characterizing categories of DCJs that can be used in optimal DCJs and indels scenarios.

In “Statement of the problem” section we define the model, give mathematical objects for genomes and rearrangement operations, from which we derive and prove some useful properties. Then we describe our algorithm in “Methods” section. We finally give the results of an implementation of our algorithm on simulated genomes, showing the limits of optimizing on indel sizes due to signal saturation, and how this optimization can improve the statistical properties of inferred DCJ scenarios. In the last section we explicit the limits of using this approach on biological data, and discuss some possible improvements on the model.

## Statement of the problem

### Genomes and DCJ

A *genome* *g* is defined as a set of *n* pairwise disjoint edges within a set of 2*n* vertices *V* (i.e., a perfect matching). A genome is *weighted* if a non negative integer weight (denoted by function *w*) is assigned to each edge, and *unweighted* otherwise. For the relation of this definition with various usual definitions of genomes in the context of rearrangements, see [2, 3, 9].

A *DCJ* is an operation on an unweighted genome transforming any pair of edges *ab* and *cd* into *ac* and *bd*. A *wDCJ* [3] acts similarly on a weighted genome, and additionally reassigns weights to the newly formed edges with the condition that *w*(*a* *c*)+*w*(*b* *d*)=*w*(*a* *b*)+*w*(*c* *d*), while the weight of the other edges remains unchanged. To any wDCJ can thus be associated an *underlying* DCJ, of which it is said to be a *weighted realization*.

An *indel* of size *δ* (where *δ* is a strictly positive integer) is an operation on a weighted genome consisting in increasing or decreasing the weight of an edge by *δ*.

### Breakpoint graph and valid scenario

Given two genomes *g*_1_ and *g*_2_ on the same vertex set *V*, we define the *breakpoint graph* as BG(*g*_1_,*g*_2_)=(*V,g*_1_∪*g*_2_). This is a 2-regular multi-graph which can be partitioned into vertex-disjoint cycles, each of even length (the length of a cycle being defined as the number of edges it contains). A cycle of length 2 (thus consisting of twice the same edge: one from *g*_1_ and one from *g*_2_) is called *trivial*. In the case of weighted genomes, a trivial cycle containing edges *e*_1_ and *e*_2_ is said to be *balanced* if *w*(*e*_1_)=*w*(*e*_2_).

**Fig. 1:**
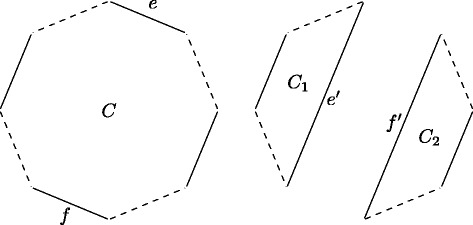
Illustration of a DCJ acting on edges *e* and *f* in a cycle *C* of a breakpoint graph. This DCJ is valid: two cycles are obtained from *C*, thus the number of cycles is increased

A DCJ (or wDCJ) on *g*_1_ or *g*_2_ is *valid* if, after it is applied, the number of cycles in the corresponding breakpoint graph is increased by one. Note that a valid DCJ (or wDCJ) necessarily acts on two edges that belong to the same cycle (see Fig. 1 for an illustration). The *DCJ-distance* [10] between two unweighted genomes is the minimum number of successive DCJs one needs to apply to *g*_1_ (or to *g*_2_) to obtain a breakpoint graph containing only trivial cycles. This distance is equal to *n*−*c* (where *n* is the number of edges in a genome and *c* is the number of cycles in BG(*g*_1_,*g*_2_)), and can be achieved by applying *any* valid DCJ at each step. Such a series of successive valid DCJs is called a *valid DCJ scenario*. We similarly define a *valid wDCJ* (or *valid wDCJ scenario*) if the underlying DCJ (or DCJ scenario) is valid. Note however that when genomes are weighted, a valid wDCJ scenario gives a breakpoint graph that is composed of *n* trivial cycles, but these may not all be balanced.

A *wDCJ scenario with indels* is a sequence of wDCJs and indels transforming one genome into the other (or, equivalently, transforming BG(*g*_1_,*g*_2_) into *n* trivial balanced cycles). It is *valid* if the underlying wDCJ scenario is valid. Its *cost* *c*(*g*_1_,*g*_2_) is the sum of the sizes of the indels it contains.

### Balancing cycles

Given a path (or cycle) *P* of a breakpoint graph BG(*g*_1_,*g*_2_), seen as a set of edges, let $w_{i}(P)=\sum _{e\in P\cap g_{i}} w(e)$ for *i*∈{1,2}. Path *P* has *imbalance* I(*P*)=*w*_1_(*P*)−*w*_2_(*P*) and *absolute imbalance* |I(*P*)|. The imbalance of a breakpoint graph I(BG(*g*_1_,*g*_2_)) is the sum of the absolute imbalances of the cycles it contains.

Given *g*_1_ and *g*_2_, a wDCJ on *g*_1_ that yields *g*1′ is called *steady* (resp. *increasing*, *decreasing*) if the imbalance of the breakpoint graph remains unchanged (resp. increases, decreases), i.e. I(BG(*g*_1_,*g*_2_))=I(BG(*g*1′,*g*_2_)) (resp. I(BG(*g*_1_,*g*_2_))<I(BG(*g*1′,*g*_2_)), I(BG(*g*_1_,*g*_2_))>I(BG(*g*1′,*g*_2_))).

### Sorting by wDCJs and indels in intergenes

We introduce the following optimization problem.

**Table:** 

SORTING BY WDCJS AND INDELS IN INTERGENES
*Instance* : Two genomes *g*_1_ and *g*_2_ defined on the same
set *V* of vertices.
*Find* : A valid wDCJ scenario with indels, whose cost
*c*(*g*_1_,*g*_2_) is minimized.

In other words, the above problem asks for a wDCJ scenario of minimum length (since it must only contain *valid* wDCJs) that, on the way, performs small indels in order to balance the intergene size. This definition is motivated by a parsimony argument: we look for a scenario minimizing the amount of genome events, especially large-scale events such as DCJs.

In the following, for ease of presentation, instead of considering wDCJ scenarios with indels that act on only one genome (e.g. *g*_1_) in order to reach the other, we actually consider wDCJ scenarios with indels acting on *both* *g*_1_ and *g*_2_, until both genomes become identical (i.e., until the breakpoint graph contains only balanced trivial cycles). This approach implicitly yields a scenario of same cost for transforming *g*_1_ into *g*_2_: first apply all wDCJs and indels for *g*_1_, in the same order, then apply all inverses of the wDCJs and indels for *g*_2_ in reverse order.

In the following, we prove that in any wDCJs and indels scenario, the minimum cost of the indels is equal to the total imbalance of the breakpoint graph, I(BG(*g*_1_,*g*_2_)). We also provide a polynomial-time algorithm that outputs a valid wDCJ scenario with indels which achieves such cost.

## Methods

In this section, we present our algorithm for sorting by wDCJs and indels in intergenes. In the following, we define a simple condition on the weights of a pair of edges in BG(*g*_1_,*g*_2_). If this condition is fulfilled, such a pair will be called *bounded*. We then prove that there always exists a pair of bounded edges in a breakpoint graph (Lemma 1), and that any valid DCJ using a bounded pair of edges can be extended into a weighted realization keeping the total imbalance of the graph unchanged (Lemma 2). Along the way, we additionally show that no valid wDCJ can decrease the imbalance of the breakpoint graph, and that any steady scenario (i.e., any scenario using steady wDCJs only) must use only bounded pairs. In other words, we give a necessary and sufficient condition for a wDCJ to belong to an optimal scenario in terms of number of DCJs plus sizes of indels.

### **Definition 1**

Consider two edges *e,f* of a breakpoint graph in the same cycle *C* and in the same genome (say *g*_1_), and let *P*_1_ and *P*_2_ denote the two paths obtained from *C* after removing *e* and *f*. Let also *W*=*w*(*e*)+*w*(*f*). Then the pair (*e,f*) is said to be *bounded* if both paths *P*∈{*P*_1_,*P*_2_} satisfy the following conditions:

$$\begin{array}{*{20}l} \text{If }\mathrm{I}(C)\geq 0:\qquad \mathrm{I}(P)&\geq -W \\ \text{If }\mathrm{I}(C)\leq 0:\qquad \mathrm{I}(P)& \leq 0 \end{array} $$

If *e,f* are both in *g*_2_, the same definition applies using −I instead of I for computing the imbalance.

We first prove that there is always a bounded pair in a breakpoint graph of two weighted genomes.

### **Lemma 1**

Let *C* be a non-trivial cycle, *e*_*m*_ be an edge of minimum weight in *C*, and *e,f* be the two neighboring edges of *e*_*m*_ in *C*. Then (*e,f*) is a bounded pair.

### *Proof*

Assume wlog that *e*_*m*_ is in *g*_2_ and *e,f* are in *g*_1_. After removing *e,f*, one obtains two paths: *P*_1_={*e*_*m*_} and *P*_2_=*C*∖{*e,f,e*_*m*_}. Let *W*=*w*(*e*)+*w*(*f*). For the first path, we have I(*P*_1_)≤0 and I(*P*_1_)≥− min(*w*(*e*),*w*(*f*))≥−*W*, since I(*P*_1_)=−*w*(*e*_*m*_). Consider now *P*_2_, and note that I(*P*_2_)=I(*C*)−*W*+*w*(*e*_*m*_). Hence, if I(*C*)≥0, we have I(*P*_2_)≥−*W*. Otherwise, since *w*(*e*_*m*_)≤*W*, we have I(*P*_2_)≤I(*C*)≤0. □

We now prove that bounded pairs can be used to perform wDCJs preserving the total imbalance of the breakpoint graph.

### **Lemma 2**

Let *g*_1_ and *g*_2_ be two weighted genomes, and consider a valid wDCJ transforming *g*_1_ into *g*1′. Then this wDCJ cannot be decreasing, and, if it is steady, then the pair of edges it is applied on is bounded. Conversely, any bounded pair of edges can be used to form a valid steady wDCJ.

### *Proof*

Let *e* and *f* be the edges used by the wDCJ, *e* ^′^ and *f* ^′^ be the two edges it creates, *C* be the cycle containing both *e* and *f*, *P*_1_,*P*_2_ be the two paths obtained by removing *e,f* from *C*, and *C*_1_=*P*_1_∪{*e* ^′^} and *C*_2_=*P*_2_∪{*f* ^′^} be the two cycles created by the wDCJ.

Clearly, the imbalance of any other cycle than *C* remains unchanged. Thus the difference in the imbalance of the breakpoint graph, *Δ*, satisfies

$$\Delta=|\mathrm{I}(C_{1})|+|\mathrm{I}(C_{2})| - |\mathrm{I}(C)| $$

Note that the imbalance can be decomposed as follows:

$$\begin{array}{*{20}l} \mathrm{I}(C) &= \mathrm{I}(P_{1})+\mathrm{I}(P_{2})+w(e)+w(f) \\ &= \mathrm{I}(P_{1})+\mathrm{I}(P_{2})+w(e')+w(f') \\ &= \mathrm{I}(C_{1})+\mathrm{I}(C_{2}). \end{array} $$

Hence *Δ*=0 if both I(*C*_1_) and I(*C*_2_) have the same sign (or one is zero), and *Δ*>0 otherwise. Thus, we already know that this wDCJ cannot be decreasing. Assume now that it is steady (i.e., *Δ*=0). Let *W*=*w*(*e*)+*w*(*f*)=*w*(*e* ^′^)+*w*(*f* ^′^). We distinguish two cases.

- If I(*C*)≥0, then I(*C*_1_)≥0 and I(*C*_2_)≥0. Hence, I(*P*_1_)+*w*(*e* ^′^)≥0 and I(*P*_1_)≥−*W*. Similarly I(*P*_2_)≥−*W*.
- If I(*C*)<0, then I(*C*_1_)≤0 and I(*C*_2_)≤0. Hence, I(*P*_1_)+*w*(*e* ^′^)≤0 and I(*P*_1_)≤0. Similarly I(*P*_2_)≤0.

In both cases, the pair (*e,f*) is bounded.

We now look at the converse case: any bounded pair of edges *e,f* can clearly be used to form a valid wDCJ. Using the same notations as before, it remains to assign weights to *e* ^′^ and *f* ^′^ to create a steady wDCJ. Let *W*=*w*(*e*)+*w*(*f*), and note that I(*P*_1_)+I(*P*_2_)+*W*=I(*C*). We consider several cases:

- If I(*C*)≥0 and I(*P*_1_)≤0, let *w*(*e* ^′^):=−I(*P*_1_) and *w*(*f* ^′^):=*W*−*w*(*e* ^′^)=*W*+I(*P*_1_). Then both quantities are positive (since by assumption I(*P*_1_)≥−*W*), I(*C*_1_)=I(*P*_1_)+*w*(*e* ^′^)≥0, and I(*C*_2_)=I(*P*_2_)+*w*(*f* ^′^)=I(*C*)≥0.
- If I(*C*)≥0, I(*P*_1_)≥0 and I(*P*_2_)≥0, then any assignment of the weights (say, *w*(*e* ^′^)=*w*(*f* ^′^):=*W*/2) satisfies I(*C*_1_)≥0, and I(*C*_2_)≥0.
- If I(*C*)≥0 and I(*P*_2_)≤0, we are in a case similar to the first one: it thus suffices to set *w*(*f* ^′^):=−I(*P*_2_) and *w*(*e* ^′^):=*W*−*w*(*f* ^′^).
- If I(*C*)<0, then I(*P*_1_)≤0 and I(*P*_2_)≤0, and I(*P*_1_)+I(*P*_2_)+*W*=I(*C*). We let *w*(*e* ^′^):= min(−I(*P*_1_),*W*) and *w*(*f* ^′^):=*W*−*w*(*e* ^′^). Then I(*C*_1_)=I(*P*_1_)+*w*(*e* ^′^)≤I(*P*_1_)+(−I(*P*_1_)), and consequently I(*C*_1_)≤0.
  We now have two cases to consider.
  - if −*I*(*P*_1_)≥*W*, then *w*(*e* ^′^)=*W*. Thus I(*C*_2_)=I(*P*_2_)+*W*−*W*=I(*P*_2_), from which we conclude I(*C*_2_)≤0.
  - if −*I*(*P*_1_)<*W*, then *w*(*e* ^′^)=−I(*P*_1_), and consequently I(*C*_2_)=I(*P*_2_)+*W*+I(*P*_1_)=I(*C*), and we also have I(*C*_2_)≤0.

In all cases, the imbalance of the two created cycles have the same sign as the imbalance of *C*, so |I(*C*)|=|I(*C*_1_)|+|I(*C*_2_)|, and the wDCJ is steady. □

By the above lemma, we know that no valid wDCJ scenario can be decreasing. Consequently, only an indel can reduce the imbalance of the breakpoint graph. We thus have the following corollary.

### **Corollary 1**

Any valid wDCJ scenario with indels between two weighted genomes *g*_1_ and *g*_2_ satisfies *c*(*g*_1_,*g*_2_)≥I(BG(*g*_1_,*g*_2_)).

We now formally introduce our algorithm that optimally solves SORTING BY WDCJS AND INDELS IN INTERGENES for two genomes *g*_1_ and *g*_2_ (Algorithm 1).



### **Theorem 1**

Algorithm 1 solves SORTING BY WDCJS AND INDELS IN INTERGENES in time *O*(*n* log*n*).

### *Proof*

We first need a straightforward sanity check on Algorithm 1. First note that applying Line 4 is always possible due to Lemmas 1 and 2 (the edges neighboring *e*_*m*_ form a bounded pair, and this pair yields a steady wDCJ). Algorithm 1 yields two scenarios transforming *g*_1_ (resp. *g*_2_) into identical genomes (as obtained when the breakpoint graph contains only balanced trivial cycles), which in turn is equivalent to outputting a single scenario from *g*_1_ to *g*_2_ with the same cost. By Corollary 1, we know that I(BG(*g*_1_,*g*_2_)) is a lower bound on the cost of any valid wDCJ scenario with indels. Moreover, during the first while loop (Lines 2–5) our algorithm produces a scenario using only steady wDCJs, hence the imbalance of any intermediate breakpoint graph is the same as the original, i.e. I(BG(*g*_1_,*g*_2_)). During the second while loop (Lines 6–8), an indel of size |I(*C*)| is performed for each imbalanced cycle, hence the total cost of indels is I(BG(*g*_1_,*g*_2_)).

Overall Algorithm 1 is correct and reaches the lower bound of I(BG(*g*_1_,*g*_2_)) for the cost of the indels, hence it is optimal.

The running time can be achieved by sorting the edges by weight once (in *O*(*n* log*n*) time), and then keeping this structure sorted through wDCJs (each wDCJ needs to read and edit a constant number of weights, with cost *O*(log*n*) each time). □

## Results and Discussion

In order to test the efficiency of our model and algorithmic result, we constructed simulated data in the following way: start with an arbitrary genome with *n*=1 000 edges, with arbitrary non negative integer weights of total sum 1 000·*n*. Perform a burn-in step of 100 000 wDCJs, such that each couple of distinct edges *ab* and *cd* is equiprobable, and transform these edges into *ac* and *bd* (or with the same probability into *ad* and *bc*). The weights of the resulting edges *ac* and *bd* are chosen by picking two random numbers *r*_1_ and *r*_2_ uniformly in resp. [0,*w*(*a* *b*)] and [0,*w*(*c* *d*)]. The abovementioned burn-in step is performed so that the weight distribution reaches an equilibrium.

Then from the resulting genome, we perform 500 wDCJs in the same way. We limited ourselves to 500 wDCJs after the starting point because it is the expected point where real scenarios stop to be parsimonious in terms of the number of DCJs. So over this point, computing parsimonious scenarios and comparing them to the real ones has less sense. Concerning the indels, between two wDCJs, we perform an indel in each edge with a certain probability *p*. We generated four sets of simulated data, one for each *p*∈{0,10^−3^,10^−2^,10^−1^}. An indel consists in picking a random number in an exponential law with mean 1, and randomly adding or retracting its integer part *δ* to the edge weight.

**Fig. 2:**
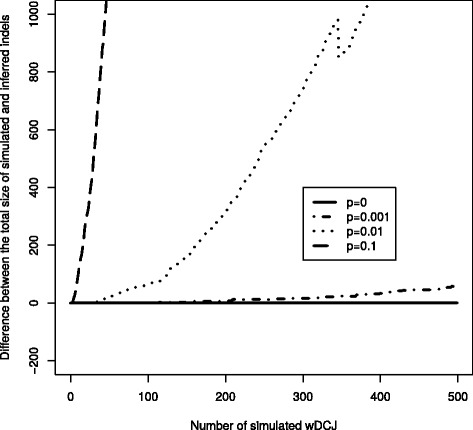
A simulation for *n*=1 000 edges, and *k*=500 successive wDCJs. The *x* axis is the step of the simulation, and the *y* axis is the difference between the real and inferred indel size

In order to evaluate the capacity of the model to infer the right indel size, we first computed, at each step, the difference between the total size of the simulated indels and the sum of the cycle imbalances, which we intepret as indels in the scenarios (see Corollary 1). The result is shown on Fig. 2. For *p*∈{0,10^−3^}, the estimations are very good (for *p*=0 there is no indel so it is just a check that our algorithm has the expected behavior). For larger *p* the signal saturates, as the number of real indels becomes quickly much larger than the number of inferred ones. This is explained by indels hitting several times the same cycle with high probability.

We then discarded the simulation with probability *p*=0.1, since the results are too divergent from the simulated numbers – in other words, our model cannot handle such an indel rate. For each of the three remaining simulations, we first computed a random DCJ scenario, consisting in picking a random valid DCJ at each step, without consideration towards the weights. We then drew a random wDCJ scenario with indels – that we will denote by *wDCJ scenario* in the following. The wDCJ scenario is constructed from a randomized variant of Algorithm 1 which works as follows: in Line 4, instead of picking a bounded pair of edges in a deterministic way, we pick a *random* bounded pair of edges, by sampling random wDCJs until one is picked that acts on a bounded pair of edges.

**Fig. 3:**
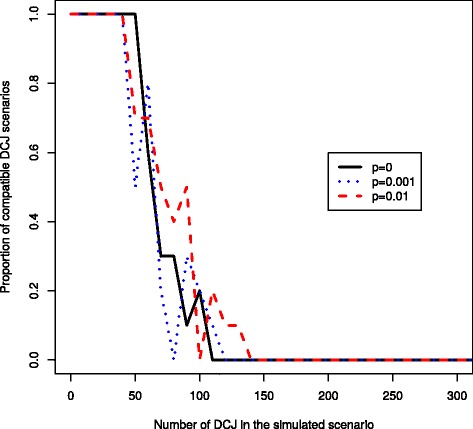
A simulation for *n*=1 000 edges, and *k*=500 successive wDCJs. The *x* axis is the step of the simulation, and the *y* axis is the proportion of random wDCJ scenarios which contain only bounded wDCJs

In order to verify first that there indeed is some signal on the scenario within intergene sizes, we computed the frequency at which a random DCJ scenario could lead to an optimal wDCJ scenario, in terms of total indel sizes. The result is shown on Fig. 3. Random DCJ scenarios, if genomes are sufficiently distant, are very improbably compatible with a scenario guided by intergene sizes. Indeed after approximately 100 wDCJs in the simulation, no scenario was using only bounded DCJs, i.e. DCJs acting on bounded pairs.

We then tested the ability of our algorithm to produce scenarios that are closer to the real ones. We measured in particular, for each vertex of the breakpoint graph (i.e., each gene extremity), how many times a wDCJ chose an incident edge in one scenario. This gives, for one scenario, a vector with one entry per vertex, and a reconstructed scenario can be compared with a real one. We could compare two scenarios by computing the sum of the squares of the differences between each vector entry.

We checked that with this measure the computed wDCJ scenario was in mean less distant to the real scenario than a random wDCJ scenario, for all simulation conditions (with a mean improvement of 4, 1, 1.7 and 2.7 *%* for *p*=0, 0.001, 0.01 and 0.1 respectively). This again argues for the existence of information on the real scenario in the intergene sizes. However the difference with random scenarios, while real, is not sufficiently spectacular to encourage us to test the algorithm on real genomic data. This would require a finer model.

## Conclusions

The contribution of this paper is twofold. First, the definition of weighted genomes [1, 3] opens combinatorial questions, one of which being the transformation of a genome into another in a minimum number of steps. In a previous paper [3] we solved the strict version of this problem, where genomes were forced to have the same total intergene sizes and only wDCJs were allowed. Here we add some flexibility to the problem, which allows all pairs of genomes to be compared, while indels are introduced to account for possible deviations in intergene sizes. Thus the present model is definitely closer to reality, where two genomes, even very close, cannot be expected to contain exactly the same total intergene sizes.

We give a polynomial solution to the distance problem, where only wDCJ optimal scenarios are allowed, and the total indel size of a scenario is minimized.

Second, this combinatorial question is related to the choice of a DCJ scenario among the many possible ones. It is a crucial question for several biological studies, about potential rearrangement hotspots for example [11]. Pevzner and Tesler [11] concluded about the existence of hotspots from the inversion distance computation, but were unable to localize them. It has even been argued that the conclusion on the existence of hotspots might depend on the choice of the scenario [12]. So it is important to exploit every available information on what might have been real scenarios. We show that some information is available in intergene sizes, by defining a necessary and sufficient property for a wDCJ to participate to an optimal scenario.

Additional work is necessary to use this information on biological data. Indeed, if there is information about the scenarios in intergene sizes, our combinatorial algorithm does not exploit it entirely. The solution space of our restricted version is still too large to propose a small number of scenarios with confidence. Statistical properties of the sub-space of solutions are only slightly closer to true scenarios than statistical properties of the whole space.

Consequently, this work opens several perspectives concerning the model, and more precisely the scoring function that should be used. One possibility is to weight indels differently. For example, one could weight an indel by an affine or an exponential function of its length. This would be biologically more relevant and would also restrict further the scenario space. Another possibility is to extend our model so as to allow other wDCJs than valid ones, i.e. wDCJs that either decrease or do not change the number of cycles in the breakpoint graph. This would allow more flexibility as, for instance, merging two unbalanced cycles may lead to a balanced one, and may thus avoid some further indels. This, however, raises the question of a good scoring function, since four events are allowed in that case (indels, and 3 types of wDCJs, depending on whether the number of cycles is decreased, unchanged or increased), and one should cleverly weight each such event.

In all cases, the study of such optimization problems, ranging from combinatorial properties to algorithmic solutions to validation of the model (via e.g. tests on simulated and real data) remains open.
